# Supplementary material for: Circulating miR-122-5p, miR-92a-3p, and miR-18a-5p as Potential Biomarkers in Human Liver Transplantation Follow-Up
Source: Int J Mol Sci. 2023 Feb 9;24(4):3457. doi: 10.3390/ijms24043457 (PMC9962619; doi:10.3390/ijms24043457)
Supplement: Supplementary file 1 [file ijms-24-03457-s001.zip › ijms-2087589-supplementary.pdf]

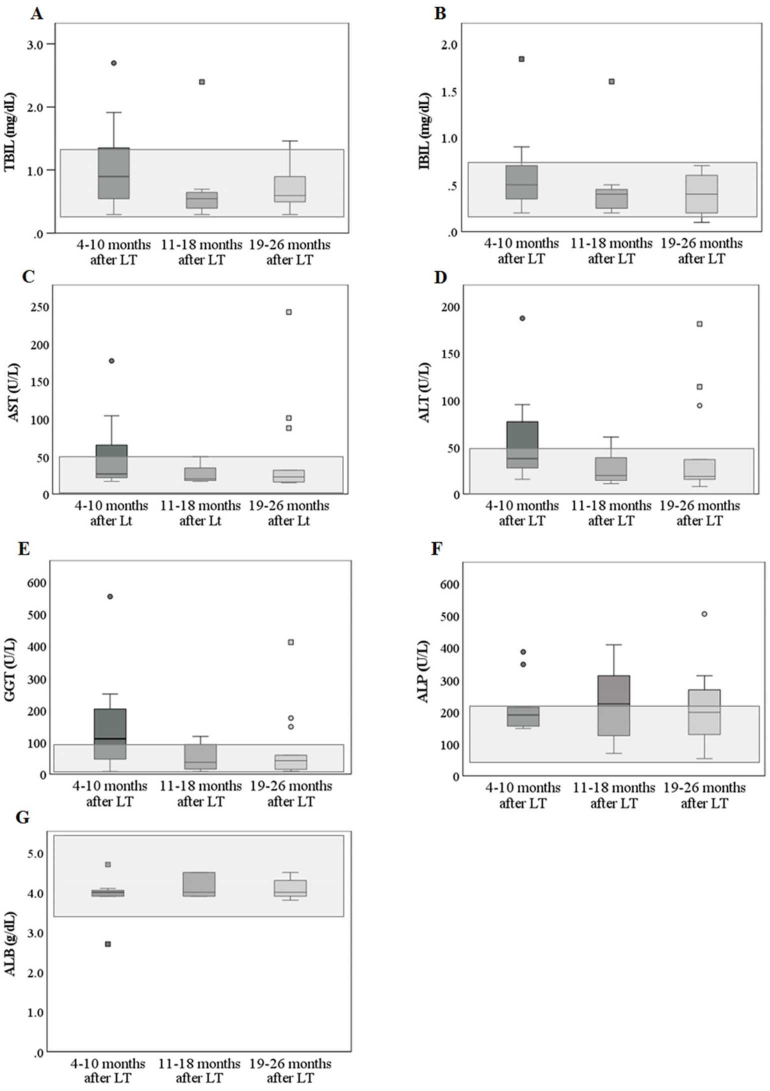

Figure S1. LFTs analyses according to FU.

LFTs were analyzed based on the FU i.e., 4-10 months (N=11), 11-18 months (N=8), and 19-26 months (N=14). Parameters are the following: total bilirubin (TBIL, panel A), indirect bilirubin (IBIL, panel B), aspartate aminotransferase (AST, panel C), alanine aminotransferase (ALT, panel D), gamma-glutamyl transferase (GGT, panel E), alpha-fetoprotein (ALP, panel F) and albumin (ALB, panel G). The grey areas indicate the LFT normal range in each graph. Nonparametric Wilcoxon test was applied but no significant differences were found along the different FU times.
